# Identification of Autophagy-Related Biomarker and Molecular Subtypes in Alopecia Areata Based on Bioinformatics Analysis, Machine Learning, and Experimental Validation

**DOI:** 10.3390/genes17060600

**Published:** 2026-05-23

**Authors:** Yufen Li, Xiaolin Zhang, Jiating Wang, Yiqun Jiang

**Affiliations:** Hospital for Skin Diseases, Institute of Dermatology, Chinese Academy of Medical Sciences & Peking Union Medical College, Nanjing 210042, China

**Keywords:** alopecia areata, autophagy, machine learning, bioinformatics analysis

## Abstract

**Background:** Alopecia areata (AA) is a common autoimmune alopecia disease. Evidence suggests that autophagy-related genes (ARGs) may contribute to its pathophysiology. This study aims to explore and identify potential autophagy-related biomarkers and molecular subtypes in AA. **Methods:** In this study, autophagy-related differential expression genes (ARDEGs) in AA were identified by comparing the differentially expressed genes (DEGs) in the GSE68801 dataset with the ARGs. Then, we applied three different machine learning methods to identify key hub genes and further verified them on independent datasets. We used the receiver operating characteristic (ROC) curve to evaluate the diagnostic potential of these hub genes and constructed a predictive nomogram. In addition, this study also used the consensus clustering method to define two AA subtypes and explored their immune characteristics and functional pathways through ssGSEA, MCPcounter and enrichment analysis. Experimental validation included qRT-PCR for four hub genes and Western blotting for critical autophagy markers. **Results:** Our analysis detected 10 ARDEGs in AA. Applying three machine learning algorithms, we identified four candidate hub genes, *ATG9B*, *EIF4EBP1*, *WIPI1* and *CCR2*, and verified their expression patterns in independent cohorts. The combined four-gene model and nomogram showed potential diagnostic performance. Consensus cluster analysis divided AA cases into two subtypes, each associated with different immune infiltration and functional pathways. Downregulation of *ATG9B* and *EIF4EBP1* and upregulation of *CCR2* were verified by qRT-PCR. Western blotting further suggested altered autophagy-related protein expression in AA lesions, characterized by a reduced LC3B-II/I ratio and Beclin-1 expression and increased SQSTM1 expression. **Conclusions:** This study identified four candidate autophagy-related genes and two exploratory molecular subtypes in AA and may provide clues for understanding autophagy-related immune dysregulation and support further validation of candidate diagnostic markers.

## 1. Introduction

Alopecia areata (AA) is a common autoimmune hair loss disorder that can affect any hair-bearing skin. Clinically, AA most commonly presents as well-demarcated patchy hair loss, but it may also manifest as extensive scalp involvement, alopecia totalis, alopecia universalis, ophiasis, sisaipho, or diffuse patterns, reflecting substantial clinical heterogeneity. Although its pathogenesis remains incompletely understood, collapse of hair follicle immune privilege (HF-IP) and subsequent autoimmune attack are considered central events. Genetic susceptibility, oxidative stress, allergy, and microbiota-related factors may also contribute to disease development [1]. Traditional treatments mainly include topical, intralesional, and systemic glucocorticoids, but recurrence, variable treatment response, and adverse effects associated with long-term glucocorticoid use remain major clinical challenges [2]. Therefore, further investigation of the molecular mechanisms underlying AA is needed to identify novel biomarkers and therapeutic targets.

**Figure 1:**
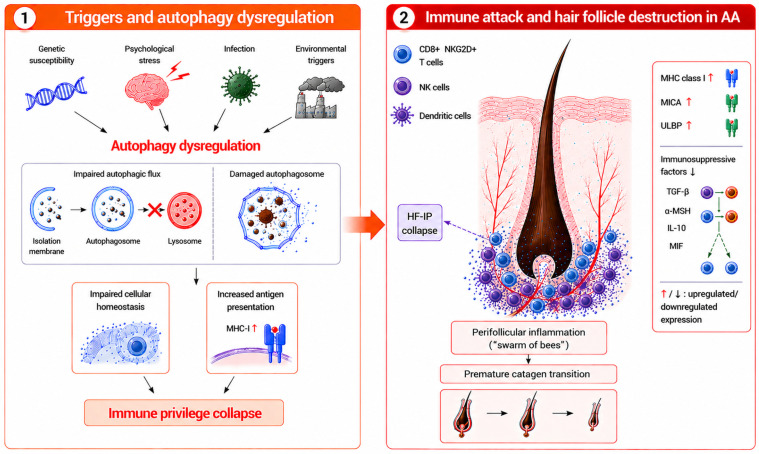
Schematic overview of AA pathogenesis and the potential role of autophagy-related alterations. AA is characterized by collapse of hair follicle immune privilege, immune-inflammatory activation, and premature transition of hair follicles from anagen to catagen/telogen phases. Genetic susceptibility and autoimmune predisposition may contribute to disease development. In this study, autophagy-related molecular alterations were identified as potential factors associated with immune dysregulation and molecular heterogeneity in AA.

Autophagy is a conserved lysosome-dependent degradation process that maintains cellular homeostasis by removing damaged organelles, misfolded proteins and other macromolecules [3]. Dysregulated autophagy has been implicated in several autoimmune and inflammatory diseases, suggesting that it may participate in immune regulation and tissue inflammation [4]. Increasing evidence also supports a potential role of autophagy in AA. Genome-wide association studies have identified AA susceptibility loci involving autophagy-related genes (ARGs), including *CLEC16A*, *STX17*, and *BCL2L11/BIM* [5,6], while copy number variations involving ARGs such as *ATG4B* and *BOK* have also been reported in AA [7]. In experimental models, IFN-γ-induced collapse of HF-IP was accompanied by impaired autophagy, whereas pharmacological modulation of autophagy partially protected against immune privilege collapse [8]. Experimental evidence from the C3H/HeJ mouse model indicated that autophagy deficiency could aggravate baldness, whereas enhancement of autophagy reduced inflammatory response in affected skin [9]. Additionally, PINK1-induced mitophagy has been reported to alleviate AA by suppressing NLRP3 inflammasome activation [10]. Overall, these findings strongly implicated that autophagy may be involved in the immune-inflammatory network of AA. A schematic overview of the potential pathogenic framework linking autophagy dysregulation and immune privilege collapse in AA is shown in Figure 1.

This study analyzed the GSE68801 dataset to systematically characterize autophagy-related differentially expressed genes (ARDEGs) in AA. Machine learning algorithms were further applied to identify key hub genes, and their expression was validated in scalp samples from AA patients and normal controls. Moreover, we investigated the association of these hub genes with molecular subtypes and immune infiltration patterns. By linking ARGs with molecular classification, immune microenvironment features, and clinical sample validation, our study provides an autophagy-focused perspective on AA and may offer new insights into the relationship between autophagy-related alterations, immune dysregulation, and disease heterogeneity.

## 2. Materials and Methods

**Figure 2:**
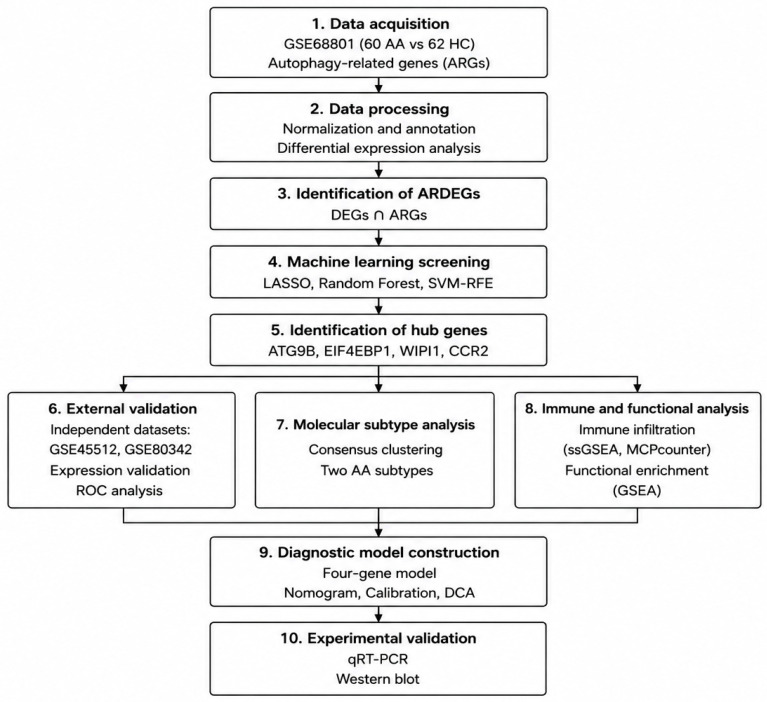
Study workflow for analyzing AA.

The study’s flowchart is illustrated in Figure 2.

### 2.1. Datasets Information and Data Processing

The gene expression data used in this study were obtained from the GSE68801 dataset in the Gene Expression Omnibus (GEO) database [11], including 60 AA lesional samples and 62 healthy control samples. A total of 222 ARGs were sourced from the Human Autophagy Database (HADb).

Raw data and series matrix files of GSE68801 were downloaded and processed in R using the “affy” package [12]. After standardization, the symbols were assigned to the genes according to the corresponding annotation file. When multiple probes were mapped to one gene, the probe with the highest average expression value across all samples was retained.

### 2.2. Characterization and Visualization of DEGs

The identification of DEGs was performed in R with the “limma” package [13]. After normalizing the raw data, logarithm conversion was carried out, and the genes that meet the set range (|log2FC| > 0.58, and adjusted *p* value < 0.05) were defined as DEGs. The Benjamini–Hochberg (BH) correction was utilized to decrease the false discovery rate (FDR). The results were then visualized by drawing heatmaps and volcano plots using “ggplot2” and “pheatmap” packages [14]. The DEGs obtained were then overlapped with 222 ARGs, and the shared genes were defined as ARDEGs.

### 2.3. Machine Learning-Based Feature Selection and Evaluation of Hub Genes

Three machine learning methods were applied for feature selection. The Least Absolute Shrinkage and Selection Operator (LASSO) regression analysis was conducted with the “glmnet” package, and the optimal penalty parameter λ was selected by 10-fold cross-validation [15]. The Random Forest (RF) model was established via the “randomForest” package for classification analysis, and RF-based recursive feature elimination was performed with internal resampling, and model stability was further assessed using out-of-bag error from a 500-tree RF model [16]. Support vector machine recursive feature elimination (SVM-RFE) was implemented with the “e1071” package, and 10-fold cross-validation was used to estimate classification error across different numbers of selected features [17]. Genes shared across all three methods were defined as hub genes.

A four-gene logistic regression model was constructed in GSE68801 and externally validated in GSE45512 and GSE80342 by applying the training-derived coefficients without refitting. The predictive performance of individual genes and the combined model was evaluated using ROC curves generated by the “pROC” package [18], and AUC values with 95% confidence intervals were calculated using DeLong’s method.

### 2.4. Defining and Confirming Autophagy-Related Clusters

We used the “ConsensusClusterPlus” package to apply the unsupervised consensus clustering method in categorizing AA patients into distinct subgroups [19]. The determination of the optimal cluster number (k) relied on a comprehensive assessment of the cumulative distribution function (CDF) curves, consensus scores, consensus matrix and silhouette width.

### 2.5. Immunological Infiltration Analysis

Two algorithms, ssGSEA and MCPcounter, were applied to evaluate the immune infiltration patterns. For ssGSEA, immune gene signatures (Immu28) were applied using the “IOBR” and “GSVA” packages [20]. Comparisons between identified clusters were conducted via the Wilcoxon test, followed by multiple-testing correction. For MCPcounter, the unscaled expression matrix was analyzed with the “MCPcounter” package to calculate the immune cell abundance scores [21]. Boxplots were generated to visualize immune infiltration differences between clusters, and significance levels were reported after FDR correction.

### 2.6. Biological Properties of Different Subtypes

To elucidate the biological mechanisms distinguishing the AA subtypes, we conducted Gene Set Enrichment Analysis (GSEA) [22]. Differential expression profiles between two clusters were compared. We performed GSEA using the “ClusterProfiler” package. The analysis employed the “c2.cp.kegg.v7.0.entrez.gmt” collection from MSigDB as the reference gene sets [23]. Gene sets with adjusted *p* value < 0.05 were considered significantly enriched.

### 2.7. Nomogram Construction and Verification

A predictive nomogram was developed and visualized based on the identified key genes using the “rms” package [24]. The model’s accuracy was evaluated by plotting a calibration curve. The clinical value of the nomogram was then evaluated using decision curve analysis (DCA).

### 2.8. Quantitative Real-Time PCR (qRT-PCR)

Total RNA was extracted from lesional scalp tissues obtained from 11 AA patients and normal scalp tissues obtained from 11 healthy controls using the RNA Extraction Kit (21023, AG, Nanjing, China). cDNA was synthesized with the SuperMix (#R323-01, Vazyme, Nanjing, China). Then, qRT-PCR was performed on a Roche LightCycler 480 system using the ChamQ SYBR qPCR Master Mix (#Q321-02, Vazyme, Nanjing, China). Gene expression levels were calculated relative to GAPDH via the 2^−ΔΔCT^ method. The sequences of all primers were detailed in Table 1.

### 2.9. Western Blot Analysis

Protein was extracted using RIPA lysis buffer. After determining protein concentration by BCA kit, samples were electrophoresed on 4–20% SDS-PAGE gels and transferred to PVDF membranes. Subsequent steps included blocking with 5% nonfat milk for 1 h and overnight incubation with primary antibodies against LC3B (#12741, 1:1000, CST, Danvers, MA, USA), SQSTM1/p62 (#5114, 1:1000, CST, Danvers, MA, USA), Beclin-1 (#3495, 1:1000, CST, Danvers, MA, USA), and β-actin (1:3000, Proteintech, Wuhan, China) at 4 °C. HRP-conjugated secondary antibody was then added, later the protein bands were detected using ECL Chemiluminescent reagent. Band intensities were quantified using ImageJ software (v1.46r) and normalized to β-actin.

### 2.10. Statistical Analysis

All statistical analyses were performed using R software (v4.2.2) and GraphPad Prism (v9.0). Data from experimental validation were presented as mean ± standard deviation. Differences between two groups were assessed using Student’s *t*-test or the Wilcoxon rank-sum test, as appropriate. Statistical significance threshold was set to *p* < 0.05.

## 3. Results

### 3.1. Identification of DEG in AA

**Figure 3:**
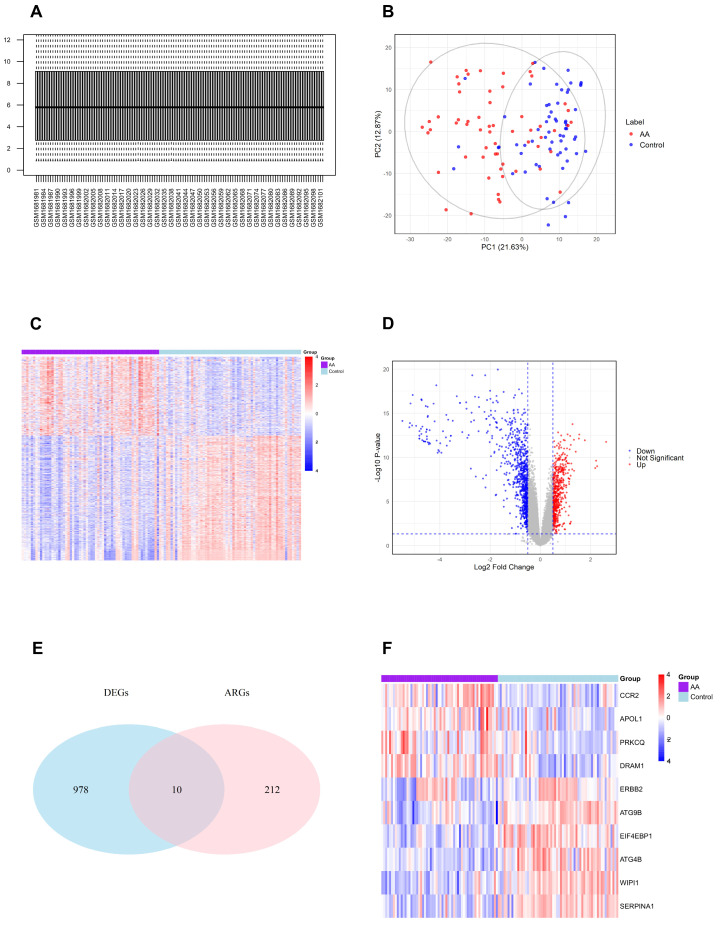
Expression pattern and screening of ARDEGs in AA. (**A**) Distribution of standardized expression values. (**B**) PCA indicating sample clustering between AA and controls. (**C**) Heatmap of DEGs between AA and controls. (**D**) Volcano plot highlighting genes with significant up- or downregulation. (**E**) Venn diagram depicting 10 overlapping genes shared by DEGs and ARGs. (**F**) Heatmap visualizing the expression differences in these ARDEGs in AA compared with normal controls.

After standardizing, all samples showed comparable expression distributions (Figure 3A). Samples from AA and control groups were distinctly separated in principal component analysis (PCA) plot (Figure 3B). Heatmap analysis revealed a distinct clustering across samples (Figure 3C), and the volcano plot highlighted 988 significantly altered genes, including 381 upregulated and 607 downregulated genes (Figure 3D). In addition, we retrieved 222 ARGs from the HADb database. By intersecting the DEGs with these ARGs, 10 ARDEGs were obtained (Figure 3E). The expression profiles of these ARDEGs in AA and control samples were presented through heatmap visualization (Figure 3F).

### 3.2. Identification of Hub Genes in AA

**Figure 4:**
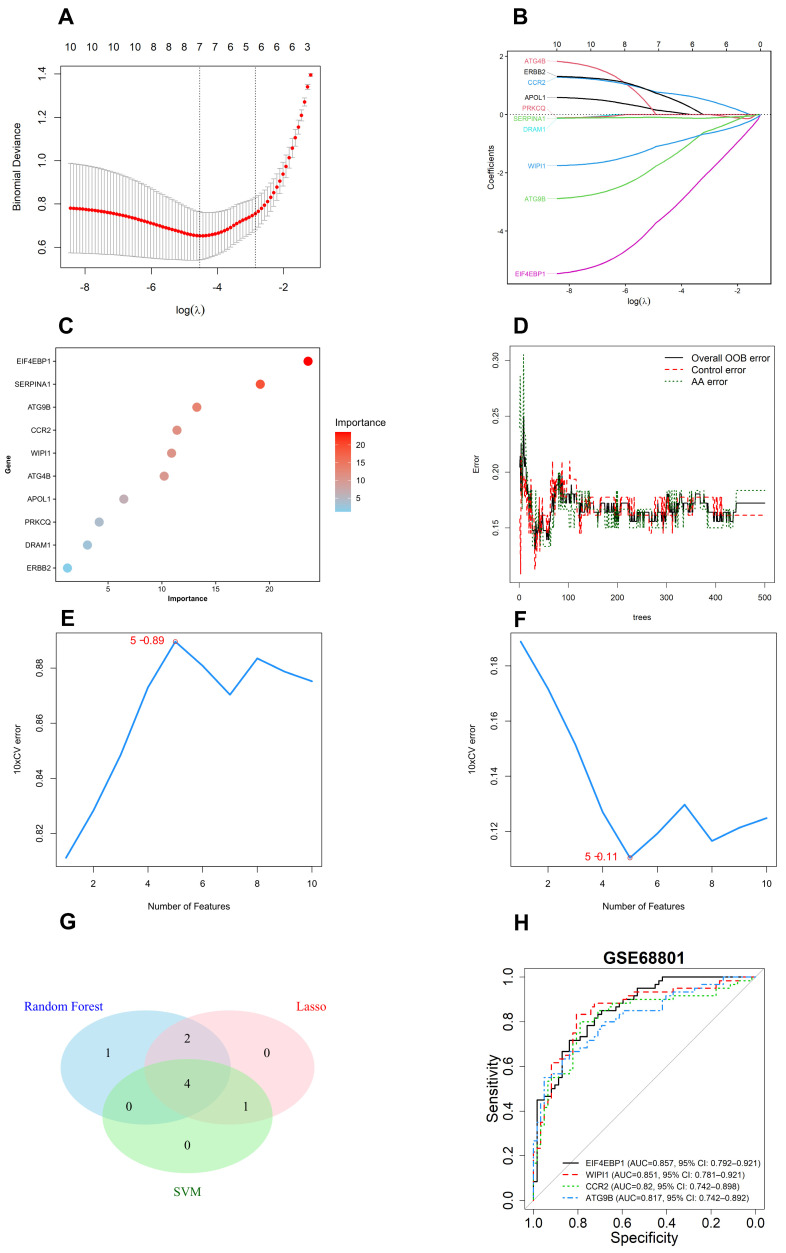
Candidate hub genes were identified through three machine learning methods: (**A**,**B**) Development of the LASSO model using 10-fold cross-validation for feature selection. (**C**,**D**) Variable importance ranking from the Random Forest algorithm. (**E**,**F**) The feature selection curve of SVM-RFE for identifying the optimal gene set. (**G**) Venn diagram identifying four candidate genes derived from three machine learning methods. (**H**) ROC curves evaluating the diagnostic relevance of the four candidate hub genes.

Hub genes were screened from the ten ARDEGs using three machine learning algorithms. The LASSO regression model, optimized through 10-fold cross-validation at lambda.min = 0.014, retained seven genes for further consideration (*EIF4EBP1*, *ATG9B*, *WIPI1*, *CCR2*, *ERBB2*, *APOL1* and *SERPINA1*) (Figure 4A,B). In parallel, an analysis with the FR method designated seven genes (*EIF4EBP1*, *SERPINA1*, *ATG9B*, *CCR2*, *WIPI1*, *ATG4B* and *APOL1*) as key diagnostic candidates, based on an importance score threshold >1.0 (Figure 4C,D). In the SVM-RFE analysis (Figure 4E,F), classification error reached its minimum when five features were included, producing another gene subset (*EIF4EBP1*, *ATG9B*, *WIPI1*, *ERBB2* and *CCR2*). The intersection of the three algorithms yielded four shared hub genes (*EIF4EBP1*, *WIPI1*, *CCR2* and *ATG9B*) (Figure 4G). ROC analysis suggested that these four candidate genes had potential diagnostic relevance for distinguishing AA from control samples (Figure 4H).

### 3.3. External Validation of the Characteristic Genes

**Figure 5:**
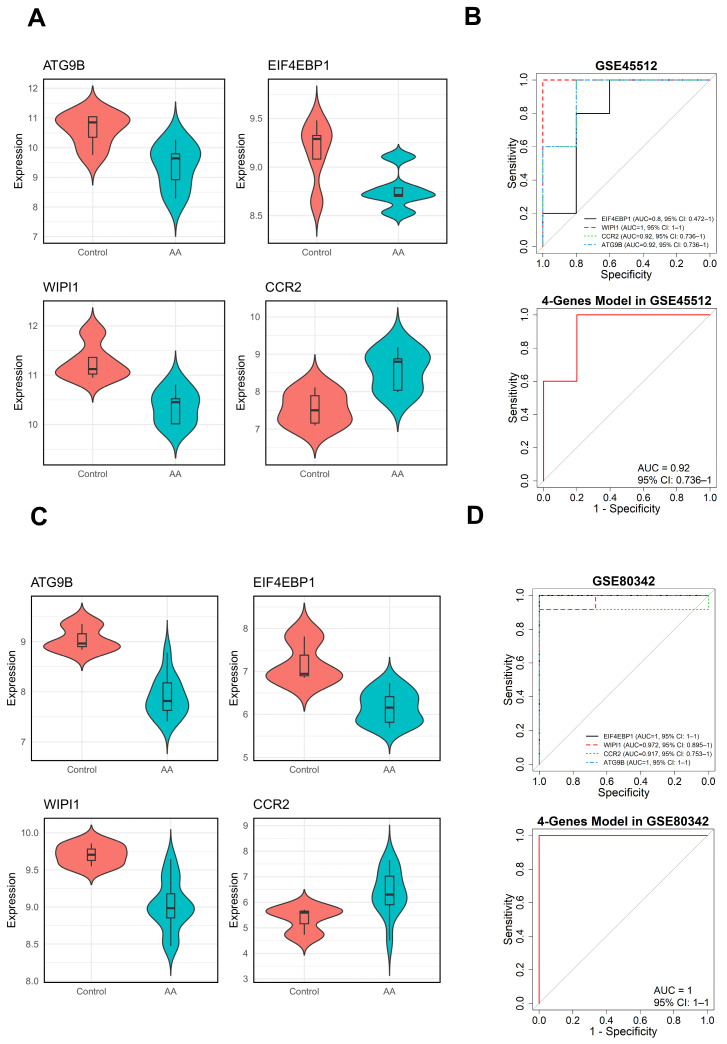
Evaluation of the four candidate genes and combined four-gene model in external datasets. (**A**) Expression distribution of the four genes shown by violin plots in GSE45512. (**B**) ROC curves of individual genes and the combined four-gene model in GSE45512. (**C**) Violin plots displaying expression changes in the four genes in GSE80342. (**D**) ROC curves of individual genes and the combined four-gene model in GSE80342.

To independently validate the identified central genes, we utilized two datasets, GSE45512 and GSE80342. Expression analysis revealed that *ATG9B*, *EIF4EBP1* and *WIPI1* were markedly downregulated in AA samples, whereas *CCR2* was significantly upregulated compared with normal controls (Figure 5A,C). ROC analysis of individual genes suggested that the four hub genes had potential diagnostic relevance. More importantly, the combined model showed improved discriminatory performance compared with individual genes (Figure 5B,D), suggesting that the selected genes may be more informative when used as an integrated autophagy-related signature. However, given the relatively limited sample sizes of the validation cohorts, further validation in larger multicenter datasets is still warranted.

### 3.4. Construction of the Diagnostic Nomogram for AA

**Figure 6:**
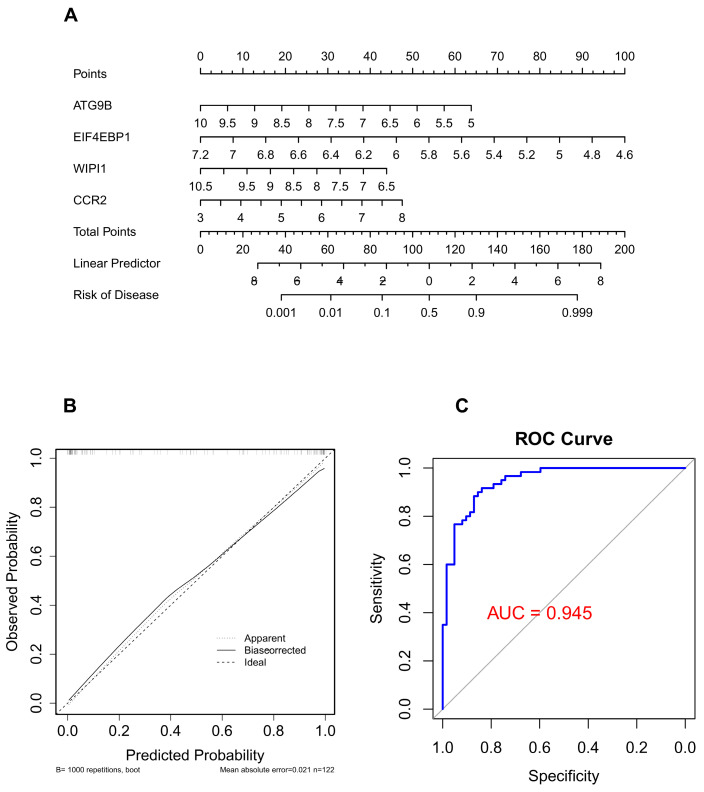
Nomogram for estimating the predicted probability of AA classification (**A**) Four candidate hub genes were included as predictor variables based on logistic regression. (**B**) Calibration curve comparing predicted and observed probabilities of being classified as AA. (**C**) The ROC curve depicts the predictive performance.

We constructed a diagnostic nomogram based on the four candidate genes to estimate the predicted probability of being classified as AA. As shown in Figure 6A, each gene contributes a specific score, and the total score corresponds to the predicted probability of being classified as AA. The calibration curve showed acceptable agreement between predicted and observed probabilities (Figure 6B) and ROC analysis suggested favorable discriminatory performance of the model (Figure 6C).

### 3.5. Consensus Clustering and Immune Infiltration Analysis

**Figure 7:**
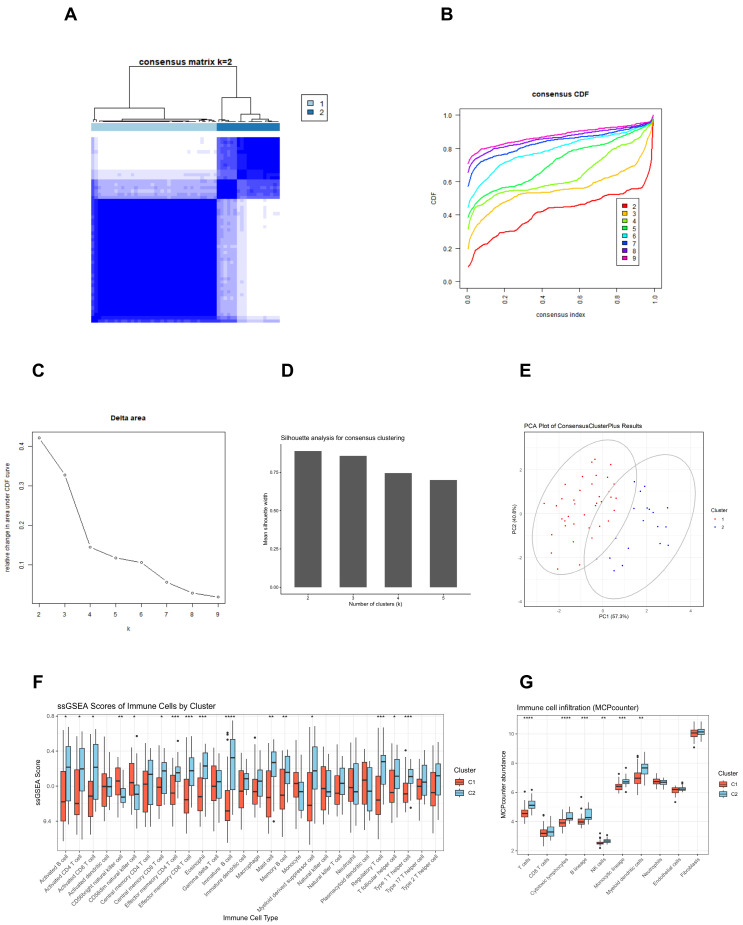
Subtype identification and immune landscape of AA based on ARGs: (**A**–**D**) Consensus clustering analysis supporting two subgroups. (**E**) PCA showing partial separation between the two clusters. (**F**,**G**) Immune infiltration patterns estimated by ssGSEA and MCPcounter. * *p* < 0.05, ** *p* < 0.01, *** *p* < 0.001 and **** *p* < 0.0001.

To explore potential molecular heterogeneity among AA patients, the “ConsensusClusterPlus” package was employed by using the expression patterns of the four candidate hub genes. The consensus matrix showed clear separation between two clusters when k = 2 (Figure 7A). The CDF curve and delta area plot indicated that increasing k beyond 2 did not substantially improve clustering stability (Figure 7B,C). In addition, silhouette analysis further supported the separation between the two clusters (Figure 7D). Accordingly, AA samples were divided into Cluster 1 (*n* = 26) and Cluster 2 (*n* = 34). PCA showed partial separation between the two clusters, suggesting distinct transcriptional patterns (Figure 7E). To characterize the immune context of these molecular subtypes, ssGSEA and MCPcounter analyses were performed (Figure 7F,G). Cluster 2 showed higher inferred enrichment of several immune cell populations, including activated T cells, macrophages, NK cells, and dendritic cells, whereas Cluster 1 displayed relatively lower immune infiltration. These findings suggest that Cluster 2 may be characterized by a relatively immune-enriched transcriptomic profile. Because clustering was performed using only four hub genes derived from feature-selection procedures, these subtype classifications should be interpreted as exploratory and hypothesis-generating rather than definitive molecular categories.

### 3.6. Biological Features of the Two Molecular Subtypes

**Figure 8:**
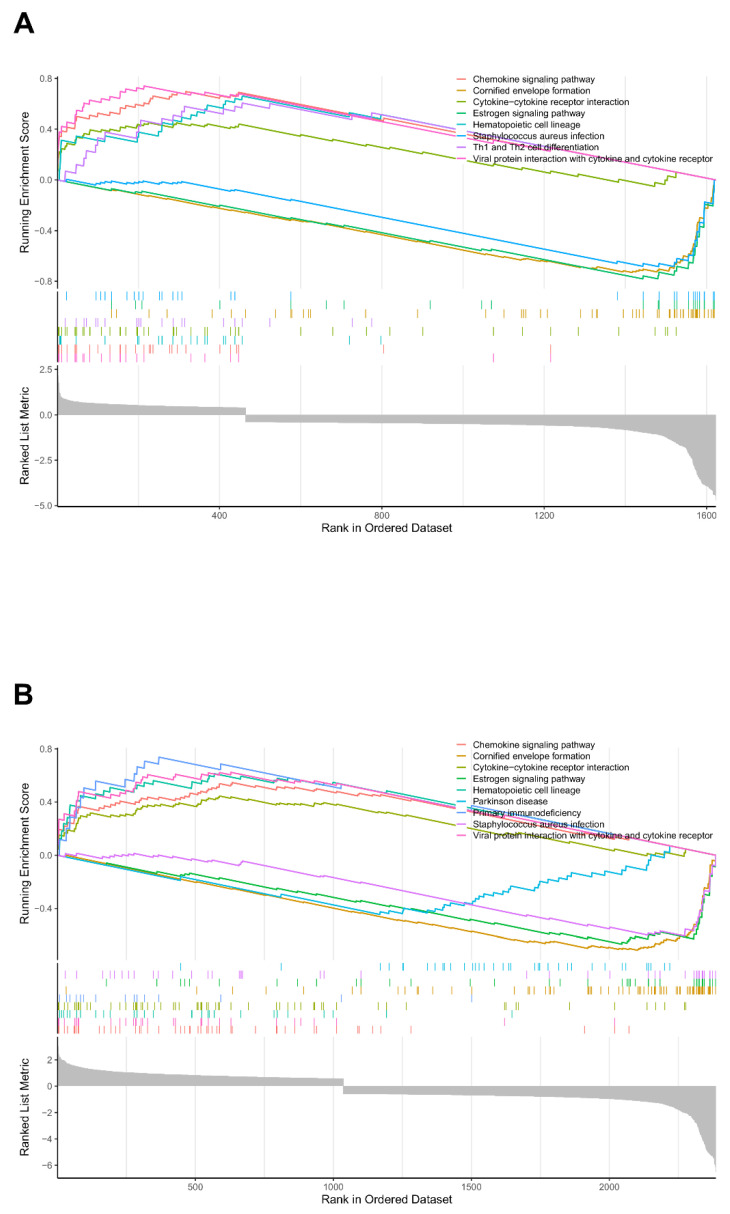
Pathway enrichment profiles of the identified subtypes: (**A**) Cluster 1 and (**B**) Cluster 2.

Gene Set Enrichment Analysis (GSEA) was further performed to characterize the biological features of the two autophagy-related clusters. Both clusters showed enrichment of immune-related pathways; however, Cluster 2 exhibited stronger enrichment of inflammatory and immune response pathways, including chemokine signaling pathway, cytokine–cytokine receptor interaction, hematopoietic cell lineage, and viral protein interaction with cytokine and cytokine receptor (Figure 8). These findings were consistent with the immune infiltration analysis, suggesting that Cluster 2 may be characterized by a relatively immune-enriched transcriptomic profile.

### 3.7. Validation of Autophagy-Related Biomarkers in Clinical Samples

**Figure 9:**
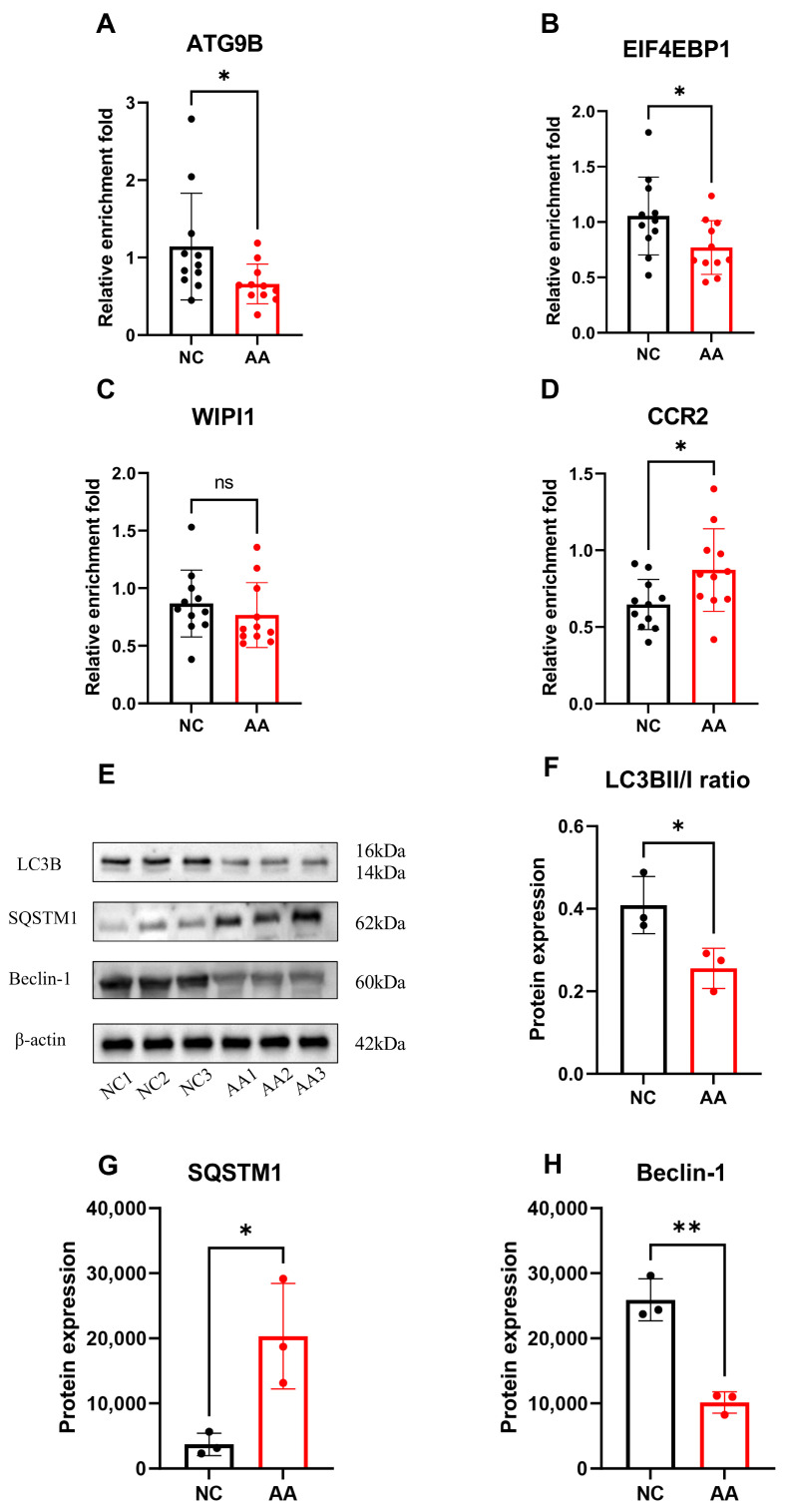
Experimental validation of four hub genes and autophagy-related proteins in AA: (**A**–**D**) mRNA expressions of four genes were quantified by qRT-PCR. (**E**) Western blot bands of LC3B, Beclin-1, and SQSTM1 in AA and healthy controls. (**F**–**H**) Immunoblot analysis of LC3B-II/I ratio, Beclin-1, and SQSTM1 levels. *: *p* < 0.05; **: *p* < 0.01; ns: non-significant.

To further validate the candidate genes in clinical samples, we measured the expression of the four ARGs and autophagy protein biomarkers in clinical samples. The qRT-PCR results revealed that *CCR2* expression was significantly elevated in AA lesions (*p* = 0.0285) (Figure 9D), while the expression levels of *ATG9B* (*p* = 0.0413) and *EIF4EBP1* (*p* = 0.0395) were significantly decreased in AA lesions compared with normal controls (Figure 9A,B). *WIPI1* showed no significant difference between two groups (*p* = 0.4194) (Figure 9C). Western blot analysis further supported altered expression of autophagy-related proteins in AA. Representative blots of LC3B, SQSTM1, and Beclin-1 were shown in Figure 9E. Quantification revealed a significant reduction of LC3BII/I ratio (*p* = 0.0351) (Figure 9E,F), a significantly decreased level of Beclin-1 (*p* = 0.003) and elevated level of SQSTM1 (*p* = 0.0257) in AA lesions (Figure 9E,G,H).

## 4. Discussion

This study analyzed gene expression profiles from the GSE68801 cohort to screen for DEGs in AA. The subsequent overlap between these DEGs and a panel of ARGs defined a set of ten ARDEGs. On this basis, four candidate hub genes were further screened using a variety of machine learning algorithms. Based on these four hub genes, consensus clustering analysis stratified AA patients into two molecular subtypes that exhibited distinct immune infiltration and biological signatures. The diagnostic value of the four candidate genes was then validated in independent datasets and further incorporated into a nomogram model. Finally, the expression differences in these core genes and autophagy-related markers were further evaluated in clinical scalp samples.

*EIF4EBP1* encodes a translational repressor regulated by mTORC1, a core hub for the integration of cellular energy status [25]. The function of the mTOR pathway is crucial for maintaining skin and hair follicle homeostasis, and its dysregulation is linked to various hair disorders, including AA [26,27]. Studies have shown that moderate mTOR inhibition can weaken local immune responses and help maintain the immune microenvironment of hair follicles [28]. These findings suggest that altered *EIF4EBP1* expression may reflect dysregulation of the mTOR–autophagy axis and may be associated with immune-inflammatory alterations in AA.

Autophagy serves as a fundamental cellular mechanism for maintaining homeostatic balance and is regulated by almost forty ARGs. Among them, *ATG9B*, as a multi-transmembrane protein, is an indispensable component in the formation and transport of autophagosome membranes [29]. Previous studies have shown that *ATG9B* was downregulated in immune-related skin diseases such as lichen planus, suggesting that impaired autophagy may interfere with T cell activation and immune regulation [30]. These data may suggest a potential link between autophagy-related dysfunction and immune regulation in AA.

The WIPI protein family (*WIPI1–4*) acts as key mediators responsible for linking the phosphatidylinositol signaling pathway with the formation of autophagosomes [31]. Previous studies have shown that abnormal upregulation of *WIPI1* can induce excessive superoxide production, thereby induce mitochondrial stress and impair its function [32]. However, the expression of *WIPI1* did not show significant differences in our samples. This discrepancy weakens the evidence supporting *WIPI1* as a stable candidate marker. Therefore, *WIPI1* should be interpreted cautiously and requires further validation in larger independent cohorts.

CCR2 is mainly expressed on the surface of monocytes, dendritic cell precursors, and some T cell subsets, and mediates inflammation recruitment by binding to CCL2. Abnormal activation of the CCL2/CCR2 axis has been confirmed to be involved in the development of multiple sclerosis, asthma, and other diseases [33]. This pathway can further activate downstream signals such as JAK2/STAT3, which are known to influence immune regulation and tissue inflammation [34]. Previous studies have shown that inhibiting CCL2/CCR2 signaling can suppress Th2-mediated immune responses and enhance the expression of Th1 cytokines, thereby reshaping local immune balance to some extent [35]. Notably, *CCR2* was upregulated in AA samples, suggesting that CCR2-related chemokine signaling may be associated with immune cell recruitment in the AA microenvironment, although its effects may vary across different immune or atopic backgrounds.

The immune infiltration and GSEA analyses suggested that the two autophagy-related clusters had distinct immune-related transcriptomic features. Cluster 2 showed relatively higher inferred immune cell enrichment and stronger enrichment of immune- and inflammation-related pathways, including chemokine signaling, cytokine–cytokine receptor interaction, hematopoietic cell lineage, and Th1/Th2-related immune processes. These findings indicate a potential association between autophagy-related alterations and immune dysregulation in AA. However, because detailed clinical metadata were unavailable, these clusters cannot be interpreted as clinically defined active, chronic, or stable disease stages. They should instead be regarded as exploratory transcriptomic subtypes requiring validation in larger clinically annotated cohorts.

Several limitations should be noted. First, the sample sizes of the external validation datasets and clinical validation cohort were relatively limited, which may affect the stability and generalizability of the findings. Second, *WIPI1* was not significantly validated by qRT-PCR, suggesting that its role should be interpreted cautiously. Third, detailed clinical data were unavailable; therefore, the identified clusters should be interpreted as exploratory transcriptomic subtypes rather than clinically defined disease stages. Moreover, the clustering analysis was performed using only four hub genes, which may limit statistical robustness and reproducibility. Larger clinically annotated cohorts and broader transcriptomic signatures are needed to validate their reproducibility and clinically confirm the stability and biological relevance of these subtypes. Finally, this study mainly provides bioinformatics evidence and expression-level validation; functional experiments are needed to determine whether the identified genes directly regulate autophagy, immune dysregulation, or hair follicle injury in AA.

## 5. Conclusions

In summary, this study identified four candidate autophagy-related genes associated with AA and explored two exploratory and hypothesis-generating molecular subtypes with distinct immune-related transcriptomic features. These findings provide preliminary evidence linking autophagy-related alterations with immune dysregulation in AA and may offer potential clues for future diagnostic and mechanistic studies.

## Figures and Tables

**Table 1:** Primer sequences used for qRT-PCR.

Gene	Forward (5′–3′)	Reverse (5′–3′)
*ATG9B*	TTCAGCGTGCGGAGGATGG	CAGAGGTGCCCAAGAAACTTAGAG
*EIF4EBP1*	AGCCCTTCCAGTGATGAGC	TGTCCATCTCAAACTGTGATCTT
*WIPI1*	TCTACAACTTGGACCCACGATG	GCAGCATAAGATGGAGGTAAGGAAG
*CCR2*	GGCATAGGCAGTGAGAGTC	CGCTTGGTGATGTGCTTCG

## Data Availability

The data are available from the corresponding author on reasonable request.
